# Predictors of Formaldehyde-related Adverse Symptoms Among Laboratory Users in a University in Malaysian Borneo

**DOI:** 10.21315/mjms-04-2025-313

**Published:** 2026-02-28

**Authors:** Muhammad Ezmeer Emiral, Sahipudin Saupin, Fatin Khairah Haron

**Affiliations:** 1 Public Health Medicine Department, Faculty of Medical and Health Sciences, Universiti Malaysia Sabah, Kota Kinabalu, Sabah, Malaysia; 2 Borneo Marine Research Institute, Universiti Malaysia Sabah, Kota Kinabalu, Sabah, Malaysia

**Keywords:** formaldehyde exposure, adverse symptoms of formaldehyde exposure, risk factors, medical students, marine science students, laboratory assistants, university laboratories

## Abstract

**Background:**

Formaldehyde exposure in university laboratories poses significant health risks to students and staff. Understanding the prevalence of adverse symptoms and associated factors is essential for guiding preventive measures. This study assessed the prevalence and predictors of formaldehyde-related adverse symptoms among students and laboratory staff at a university in Malaysian Borneo.

**Methods:**

This cross-sectional study employed a validated self-administered questionnaire capturing sociodemographic characteristics, medical and allergy history, exposure frequency, and control measures. Data were analysed using bivariable logistic regression and multivariable logistic regression to identify independent predictors.

**Results:**

Among 157 respondents, 34.4% (95% confidence interval [CI]: 27.0, 42.4) reported formaldehyde-related adverse symptoms. The most common complaints were headaches/dizziness (19.1%), unusual tiredness or drowsiness (12.1%), and stress/irritability (7.0%). Five independent predictors were identified: female gender (adjusted odds ratio [aOR]: 3.56; 95% CI: 1.26, 10.10), age ≥ 30 years (aOR: 9.08; 95% CI: 2.33, 35.35), and a history of allergy (aOR: 2.80; 95% CI: 1.09, 7.25). Higher exposure frequency markedly increased symptom risk, with fortnightly (aOR: 7.76; 95% CI: 2.31, 26.09) and weekly formaldehyde handling (aOR: 3.32; 95% CI: 1.05, 10.52) associated with significantly higher odds than monthly exposure. Face mask use showed a strong protective effect (aOR: 0.04; 95% CI: 0.01, 0.10).

**Conclusion:**

Given the high prevalence of formaldehyde exposure symptoms, particularly among older individuals, women, individuals with allergy, and frequent handlers, strengthening control measures—particularly consistent mask use and reduction of exposure frequency—should be prioritised to minimise risks in university laboratory settings.

## Introduction

Formalin is commonly used in medical and veterinary schools for preserving cadavers and biological specimens, but its potential toxicity in dissection halls is often overlooked (1). In medical schools, a cadaver is preserved by infusing chemical substances, including formalin, carbolic acid, alcohol, dye, and glycerine, through the internal carotid or femoral arteries (2). Formaldehyde fumes released from preserved cadavers and preservation fluids can adversely affect the health of instructors, embalmers, anatomists, laboratory personnel, and medical, dental, and veterinary students. Reported effects include irritation, immunologically mediated sensitisation, and carcinogenicity (3). Common acute symptoms include headache, upper respiratory tract irritation, skin irritation, and eye irritation (4, 5). Formaldehyde has been demonstrated to cause nasal squamous cell carcinoma in experimental rats and irritate the respiratory tract and ocular mucosa. Accordingly, many international environmental protection agencies list formaldehyde as a possible human carcinogen (5, 6).

Occupational formaldehyde exposure is linked to increased systemic health impairments (7). Formaldehyde effects vary by anatomical location and dose, notably impairing the respiratory system. Nevertheless, comprehensive studies examining its impact on diverse organ systems and reversal/protective mechanisms are lacking. The high solubility of formaldehyde in water leads to rapid absorption in the respiratory and gastrointestinal tracts (8). Once absorbed, formaldehyde is rapidly broken down into formate, which can then be oxidised to and exhaled as carbon dioxide or incorporated into biological matrices. Formate can be measured in blood and urine and has been proposed as a biomarker of formaldehyde exposure (7). However, biological monitoring of formaldehyde exposure is not a common practice.

Formalin is widely used in all public university laboratories in Malaysia. Because of its necessity for tissue specimen preservation, it is inevitable in the daily workflow of a laboratory. Despite this widespread use, to date no study has investigated the adverse effects of formaldehyde exposure among students and personnel working directly with formalin in university laboratories, particularly in Sabah, a state in Malaysian Borneo. Sabah has one of the largest public higher learning institutes in Malaysia, with numerous medical students and laboratory staff in the Faculty of Medicine and Health Science and the Borneo Marine Research Institute. These populations are routinely exposed to formalinised human/animal cadavers and biological specimens, highlighting the need to ascertain the exposure level and associated health effects in this vulnerable population.

In this study, we investigated the prevalence of adverse symptoms related to formaldehyde exposure among the most vulnerable group in the laboratories of a large research institute in Sabah, Malaysia. The specific objectives were to: i) determine the prevalence of self-reported symptoms of formaldehyde exposure among laboratory assistants and students in research institute laboratories, and ii) identify the factors influencing formaldehyde exposure and symptom occurrence.

## Methods

### Research Design and Study Location

This cross-sectional study investigated formaldehyde exposure and related adverse symptoms among students and laboratory personnel in university laboratories in Sabah, Malaysian Borneo. Data collection was performed from February to April 2023. Respondents, including students and laboratory staff, completed a questionnaire assessing adverse symptoms associated with formaldehyde exposure after their respective laboratory classes or work shifts.

This study was conducted in six university laboratories in Sabah, Malaysian Borneo: an Anatomy Lab located in the Faculty of Medicine and Health Sciences and five laboratories in the Borneo Marine Research Institute (Physical Oceanography, Fish Disease Studies, Algae Culture and Studies, Tropical Ecosystems, and Biotechnology). These laboratories were selected based on their involvement in formalin handling and the presence of a larger number of students and staff members.

### Study Population and Eligibility Criteria

The study population comprised first-year medical students. laboratory assistants (including laboratory technologists, science officers, assistant science officers, and general assistants), and fourth-year undergraduate and master’s marine science students from the aforementioned institutes and laboratories. At the time of writing, the total population in the research institutions was 216 individuals: five laboratory assistants and 102 first-year medical students from the Faculty of Medicine and Health Sciences, as well as 24 laboratory assistants and 85 marine science students from the Borneo Marine Research Institute.

We included only first-year medical students, as others no longer participate in anatomy classes or dissections. Similarly, first-, second-, and third-year undergraduate marine science students were excluded as the majority did not engage in laboratory practicals or experiments. Cleaners were also excluded from the study, as they do not directly handle formalin and are present in the laboratory for only short periods.

### Sampling and Sample Size Calculation

The respondents were selected using purposive sampling—a method considered effective due to the limited number of individuals who can serve as primary data sources, considering study design and objectives.

Sample size was calculated using the formula described by Lemeshow et al. (9), by selecting the highest prevalence of adverse reported symptoms (71.3%) from a previous study involving formaldehyde exposure among medical students and staff in an anatomy laboratory (10), with a desired margin of error of 0.05 and a 95% confidence interval (CI). On the basis of these parameters, the minimum sample size required was 129. To account for potential missing data and nonresponse rates, the final sample size was increased by 30% to 184 respondents.

### Research Instrument and Administration

The questionnaire used in this study was based on validated questionnaires on formaldehyde exposure that had been previously used in studies conducted in university and hospital laboratory settings (4, 10). The questionnaire had undergone thorough design and testing to ensure the collection of accurate and reliable information about formaldehyde exposure and associated symptoms as per the author.

Respondents independently completed a questionnaire that included sections on demographic details, medical history, occupational characteristics, and formaldehyde exposure. Furthermore, inquiries about symptoms experienced during working hours as well as outside of working hours, which may have been linked to formaldehyde exposure were recorded. Respondents first indicated if they experienced each symptom during work (Q1). If yes, they were then asked if it was related to the workplace (Q2) and whether the symptom improved when away from work (Q3; e.g., weekends and holidays). A symptom was considered “work-related” if the respondent answered “Yes” to all three items (Q1–Q3). Throughout the administration of the questionnaires, the principal investigator was present to address any queries or concerns raised by the respondents regarding the questionnaire or its content to help them provide accurate responses.

### Ethical Approval, Informed Consent, and Data Management

Ethical approval for this study was obtained from the Medical Research Ethics Committee of Universiti Malaysia Sabah (approval ID: JKEtika 1/23 [23]); approval date: 24 March 2023. Thereafter, all potential respondents were contacted and provided with study details and objectives. A list of students and laboratory assistants was obtained from class representatives and respective laboratory supervisors. Those who expressed consent to participate were provided with a link to the questionnaire in Google Forms and were requested to complete and submit the online questionnaire on the same day that they engaged in tasks involving formalin. Before starting the questionnaire, respondents’ consent was recorded by clicking “I agree” on the form. This ensured that individuals who did not provide their consent were automatically excluded from the study. The questionnaire was estimated to take approximately 10 to 15 min to complete.

The researchers had periodic access to monitor the responses. The respondents were asked to provide their mobile phone number, email address, and personal contact details, which were stored in a secure password-protected database. This information was not linked to a study ID number for this research, thereby ensuring data confidentiality. Each respondent was assured that their data would be treated with strict confidentiality and used solely for research purposes, as well as that their anonymity would be preserved. All data recorded were kept confidential and accessible only to the researchers.

All data were analysed using IBM SPSS Statistics, Version 25 (IBM Corp., Armonk, NY, USA). Descriptive statistics were used to summarise the respondents’ sociodemographic data, such as age, gender, alcohol use, smoking habits, and body mass index. Sociodemographic data; medical history variables, such as allergies, chronic lung disease, and other comorbidities; and occupational history variables, such as frequency, duration, job title, and hazard control in place or practice, were categorised dichotomously as either “Yes” or “No.” The dependent variable was a binary outcome coded as “Yes” or “No.” A “Yes” outcome was assigned if the respondent reported at least one symptom during work (Q1), considered it work-related (Q2), and indicated that it improved when away from work (Q3). A “No” outcome was assigned if these criteria were not met.

Simple (bivariable) binary logistic regression was first conducted to examine the association between each potential predictor and formaldehyde exposure symptoms. Variables with *P* < 0.25 in the bivariable analysis and those considered clinically important were input into the multivariable model, following the purposeful selection approach described by Hosmer and Lemeshow. Eight predictors met these criteria at the screening stage: gender, age group, allergy history, preexisting illness, frequency of formaldehyde handling, exposure duration, exhaust fan availability, and face mask use.

To obtain a parsimonious model while accounting for the limited number of outcome events (*n* = 54), we performed stepwise exploratory analyses using forward and backward likelihood ratio (LR) procedures. Both approaches consistently identified the same subset of five predictors: gender, age group, allergy history, frequency of exposure, and face mask use. Because glove use was almost perfectly correlated with face mask use, only face mask use was retained in the multivariable model to avoid multicollinearity. The five predictors were subsequently entered simultaneously using the enter method to obtain the final main effects model and adjusted odds ratios (aOR) with 95% CIs. Multicollinearity and interaction checks were performed. Pairwise correlations between predictors were all < 0.6, indicating no problematic multicollinearity. Potential interaction terms (age × frequency and gender × allergy) were examined but did not improve model fit and were not statistically significant; therefore, only main effects were retained in the final model. Model fit and discrimination were evaluated using the Hosmer–Lemeshow goodness-of-fit test, classification accuracy and the area under the receiver operating characteristic (ROC) curve.

## Results

### Response Rate and Respondents’ Sociodemographic, Medical, and Occupational Characteristics

We sent out questionnaires to 184 eligible individuals; of them, 157 valid responses were obtained (response rate: 85.3%). Among them, 67.5% were from the anatomy laboratory in the Faculty of Medical and Health Science; the remaining were from Borneo Marine Science laboratories, comprising Biotechnology (4.5%), Tropical Ecosystem (9.6%), Fish Disease Research (5.6%), Algae Culture (5.1%), and Physical Oceanography (5.7%) (Table 1). There were more female respondents (60.5%) than male respondents (39.5%). Most respondents were nonobese (90.4%). The predominant age group was < 20 years (50.3%), followed by 21 to 29 years (31.2%) and ≥ 30 years (185%). In terms of ethnicity, most respondents were Bumiputera Sabah (58.5%) and Malays (18.4%). A small proportion of the respondents reported consuming alcohol (16.5%), while an even smaller proportion affirmed being smokers (8.2%). The prevalence of self-reported allergies differed across laboratories, with the highest in the anatomy laboratory (20.4%), followed by the Tropical Ecosystem Laboratory (4.5%), Biotechnology Laboratory (3.2%), Fish Disease Research Laboratory (2.5%), and Algae Culture Laboratory (1.3%). Preexisting illness (defined as receiving treatment for chronic medical conditions such as diabetes mellitus, hypertension, dyslipidaemia, asthma, and cancer) was reported by 25.5% of respondents. Most respondents were students (83.4%), thereby reflecting in the year of experience as most (56.1%) had less than one year of experience, particularly in the anatomy laboratory.

### Prevalence of Self-reported Symptoms Related to Formaldehyde Exposure

Fifteen self-reported symptoms related to formaldehyde exposure were assessed. A respondent who reported at least one of the 15 symptoms related to work activity and whose symptoms improved when away from work was considered positive for adverse symptoms of formaldehyde exposure.

Table 2 provides the overall prevalence of adverse symptoms of formaldehyde exposure in this study based on the defined criteria among the respondents. Of 157 respondents, 54 (34.4%) reported having at least one of the 15 adverse symptoms of formaldehyde exposure, whereas 103 (65.6%) did not report such symptoms. The prevalence of self-reported symptoms relating to formaldehyde exposure ranged from 0.6% to 19.1%, and the most common symptom was dizziness or headache (19.1%), followed by unusual tiredness, fatigue, or drowsiness (12.1%). Other symptoms were stress or irritability (7.0%), eye irritation (6.4%), neck or shoulder pain/stiffness (5.7%), itchy skin (5.1%), shortness of breath (3.2%), and difficulty remembering or concentrating (3.2%). Other symptoms were reported in < 2.0% of the respondents.

### Association between Respondents’ Characteristics and Formaldehyde Exposure Symptoms

Table 3 presents the results of the simple (bivariable) logistic regression. Female respondents had significantly higher odds of experiencing adverse symptoms of formaldehyde exposure than males (odds ratio [OR]: 2.49; 95% CI: 1.21, 5.13). Respondents aged ≥ 30 years had nonsignificantly higher odds of reporting symptoms of formaldehyde exposure than those aged ≤ 29 years (OR: 1.38; 95% CI: 0.55, 3.48; *P* = 0.49). However, having an allergy history increased the likelihood of experiencing adverse symptoms (OR: 2.2; 95% CI: 1.11, 4.30). Respondents with preexisting illness referred to chronic medical conditions (e.g., diabetes mellitus, hypertension, dyslipidaemia, asthma, cancer), also had higher odds of adverse symptoms of formaldehyde exposure (OR: 2.12; 95% CI: 1.02, 4.43). Other factors examined, including alcohol consumption, smoking status, job title, and faculty/institute, were not significantly associated with adverse symptoms.

### Association between Hazard Control Practised and Adverse Symptoms of Formaldehyde Exposure

Compared with monthly formaldehyde handling/exposure, more frequent handling—fortnightly (OR: 3.62; 95% CI: 1.59, 8.25) or weekly (OR: 2.475; 95% CI: 1.05, 5.78)—was significantly associated with higher odds of experiencing adverse symptoms (Table 4). Exposures lasting 1 to 2 h (OR: 1.65; 95% CI: 1.23, 5.71) and > 3 h (OR: 5.23; 95% CI: 1.82, 15.05) were significantly associated with higher odds of adverse symptoms compared with exposures lasting < 1 h.

The presence of an exhaust fan (OR: 0.499; 95% CI: 0.25, 0.97), glove use (OR: 0.11; 95% CI: 0.05, 0.23), and face mask use (OR: 0.07; 95% CI: 0.03, 0.15) were significantly associated with lower odds of experiencing adverse symptoms. The presence of a fume hood, biosafety cabinet, or localised ventilation was not significantly associated with symptoms.

### Multivariable Analysis

Eight variables met the bivariable screening threshold (*P* < 0.25) and were considered for multivariable modelling. Exploratory forward and backward LR procedures consistently retained the same five variables, which were then included simultaneously using the enter method in the final adjusted model.

In this adjusted model (Table 5), female respondents had significantly higher odds of experiencing symptoms of formaldehyde exposure than male respondents (aOR: 3.56; 95% CI: 1.26, 10.10; *P* = 0.017). Respondents aged ≥ 30 years had markedly higher odds than those aged ≤ 29 years (aOR: 9.08; 95% CI: 2.33, 35.35, *P* = 0.001). A history of allergy was independently associated with symptoms (aOR: 2.80; 95% CI: 1.09, 7.25; *P* = 0.033). A clear exposure–response relationship was noted for frequency of formaldehyde handling: compared with monthly handling, fortnightly (aOR: 7.76; 95% CI: 2.31, 26.09; *P* < 0.001) and weekly (aOR: 3.32; 95% CI: 1.05, 10.52; *P* = 0.041) handling significantly increased the odds of symptoms. Face mask use remained strongly protective (aOR: 0.04; 95% CI: 0.01, 0.10; *P* < 0.001).

The final model demonstrated a good fit (Hosmer–Lemeshow *P* = 0.263) and excellent discrimination (AUC = 0.885).

#### Model Statistics

Hosmer–Lemeshow χ^2^ = 10.035 (*df* = 8), *P* = 0.263

Classification accuracy = 82.8%

AUC = 0.885 (95% CI: 0.830, 0.940)

## Discussion

The overall prevalence of symptoms related to formaldehyde exposure in the participating institutional laboratories was 34.4%. Consistently, international studies have demonstrated a high prevalence of symptoms among students and laboratory staff in various institutions, although reported prevalence varies considerably, ranging from 35% in southern Italy to 77% in Nigeria and 68% in Egypt (11–13). Notably, we considered symptoms as work-related only when they met all of the following three criteria: occurring during working hours, considered related to workplace exposures, and improved when being away from work. This means that our findings represent acute symptoms during work with recovery away from the laboratory. The use of a “better away from work” parameter through questions is standard in occupational case histories for work-related asthma and rhinitis and is recommended in clinical statements and position papers (14). In formaldehyde-exposed workers, the Agency for Toxic Substances and Disease Registry toxicological profile reported that approximately two-thirds experienced symptom improvement away from work, consistent with our criterion (15).

Among the different laboratory settings, respondents from the anatomy and pathology laboratories reported the highest prevalence of symptoms of formaldehyde exposure. This is likely attributable to more frequent involvement in activities requiring formaldehyde use, particularly during cadaver preservation and pathological sample preparation (6). For instance, histological workers from Malaysian universities were reportedly exposed to formaldehyde concentrations > 140% higher than those measured among administration staff (4). Such working conditions have also been associated with high exposure levels, which may exceed the NIOSH standard of 0.016 ppm and increase carcinogenic risk (5). Although exposure concentrations were not directly measured in the current study, the results indicate the need to address the high formaldehyde exposure levels and mitigate the risk of associated adverse health outcomes.

In our study, the symptoms associated with formaldehyde exposure were categorised based on different bodily systems, with neuropsychiatric symptoms such as dizziness/headache, unusual tiredness, fatigue, or drowsiness, and stress/irritability (7.0%) accounting for the highest prevalence. These findings are consistent with studies from Malaysia and Libya in which headache is commonly reported by anatomy laboratory assistants following formaldehyde exposure (4, 6). Similarly, a study conducted in the Department of Human Anatomy at a Nigerian university discovered that approximately 77% of students experienced significant discomfort due to formaldehyde exposure (12). Formaldehyde exposure can affect the central nervous system and interact with various biological processes, leading to neurological and psychiatric symptoms, such as dizziness, headache, and impaired cognitive function (16, 17).

Several studies conducted in university laboratory settings have reported that the most common clinical symptoms of formaldehyde exposure were ocular and upper and lower respiratory symptoms (12). For instance, postgraduate medical students, specialists, and technicians from an Italian teaching hospital reported symptoms such as olfactory sensitivity; eye, nose, and throat irritation; coughing; nasal congestion; and chest discomfort following formaldehyde exposure (11).

Our findings align with those of prior studies reporting that exposure to mixed solvents causes neurophysiological and psychological disorders. The neurotoxicity of formaldehyde may share similar mechanisms with other mixed solvents, such as toluene and xylene, which are commonly found in a range of products such as paints, glues, adhesives, coatings, and degreasing agents (18). Organic solvents, including combinations thereof, can cause neurotoxic consequences in exposed workers (19). The National Institute for Occupational Safety and Health lists neurotoxic illnesses caused by solvent exposure as one of the top 10 work-related diseases and injuries. With the predicted growth in the production and use of organic solvents, many of which may have neurotoxic qualities, the number of exposed individuals, especially laboratory personnel, is likely to increase lest proactive precautions are implemented.

We also observed an association between gender and symptoms of formaldehyde exposure, with a higher proportion of women meeting the symptom criteria and reporting more severe clinical manifestations of formaldehyde exposure. This finding warrants consideration in the context of potential gender-related differences in symptom manifestation and susceptibility. Although direct evidence linking gender differences to formaldehyde exposure remains limited, some studies have suggested gender-related differences in how response to formaldehyde exposure (20). Compared with men, women have a higher frequency of autoimmune and inflammatory illnesses, stronger B- and T-cell-mediated immune responses, and greater antibody responses to diverse foreign antigens (21). Other contributing factors may be due to indirect gender-related differences in precautionary measures, such as willingness to participate in training on the use of formaldehyde and respiratory personal protective equipment (PPE) in the laboratory (22). However, further research is required to explore and confirm these associations, as factors such as individual sensitivity, exposure duration, and concentration levels of formaldehyde may also contribute to the symptoms.

Age ≥ 30 years also remained a significant independent predictor in the multivariable model. Several mechanisms may explain this association. Ageing is accompanied by reduced mucociliary clearance, decreased airway epithelial thickness, and diminished antioxidant defences, all of which can impair airway protection and increase susceptibility to irritants, such as formaldehyde (23, 24). In addition, older adults generally have longer cumulative exposure from occupational and domestic sources and a higher prevalence of preexisting respiratory or allergic conditions, both of which may amplify the effects of irritant gases (25). Studies involving exposure to chemical fumes and aldehydes have likewise reported a higher prevalence of irritant symptoms among older staff and technicians (26).

Another factor associated with a higher risk of formaldehyde-related symptoms was a history of allergies, consistent with findings from a study conducted among first-year medical students in Poland (27). Individuals with allergic conditions are characterised by higher sensitivity to toxic substances and lower tolerance threshold to formaldehyde concentrations, which may lead to more rapid symptom onset (28). In some particularly sensitive people, mucosal irritation may induce histamine-mediated hypersensitivity reactions (29). This may explain the symptoms reported by allergic laboratory assistants and students in the present study. Similarly, medical students with commodities such as atopic dermatitis and allergic rhinitis have been reported to be more susceptible to formaldehyde exposure and more likely to develop mucocutaneous symptoms (30). A recent systematic review and meta-analysis also noted that each 10 μg/m^3^ increase in formaldehyde exposure was significantly associated with a 10% increase in asthma risk (31). Such clinical manifestations developed probably due to the impaired barrier function and remodelling of the skin and mucosa (30). Thus, exposure to gaseous formaldehyde, even at low concentrations, may exacerbate common atopic symptoms, particularly in individuals with chemical sensitivity (32).

In this study, exposure frequency demonstrated a clear dose–response effect: respondents who handled formaldehyde weekly or fortnightly had significantly higher odds of symptoms than those who handled it monthly. This finding is consistent with prior studies reporting significant relationships between total formaldehyde exposure duration and the development of skin, eye, respiratory, and neurological symptoms (33, 34). The molecular basis for this dose–response relationship may be linked to cumulative effects on synaptic proteins; for instance, repeated formaldehyde inhalation has been shown to impair olfactory function by decreasing SNAP25 protein levels in the olfactory bulb and synaptosome (35). Such molecular changes suggest that individuals may experience a diminished ability to perceive the chemical, leading to “olfactory fatigue.” In this state, individuals stop noticing the pungent odour even when hazardous levels are still present, potentially resulting in prolonged accidental exposure. Similarly, Wei et al. (36) noted that symptom severity was associated with time spent in the anatomy laboratory, indicating that decreasing exposure duration may ameliorate the symptoms. Nonetheless, formaldehyde remains a sensitiser, and continued low-level exposure may still trigger clinical manifestations of varying severity.

We observed a significant influence of PPE use on formaldehyde exposure symptoms. In the adjusted model, the use of face masks demonstrated a strong independent protective effect in the adjusted model, reducing the odds of symptoms by approximately 96%. Face masks, particularly respirators with chemical cartridges, provide inhalation protection by filtering out gas and vapour such as formaldehyde particles, whereas gloves act as a physical barrier against direct skin contact and absorption (37, 38). Consistent with our findings, Tiruneh (1) reported that most anatomy students and staff members who did not use PPE had a high prevalence of acute formaldehyde exposure symptoms, such as unpleasant smells (72.7%) and headache (25.0%), whereas Alnagar et al. (6) concluded that wearing masks minimised many symptoms (e.g., mouth dryness, respiratory distress, and prolonged sleeping time) among students in gross anatomy laboratories. These findings underscore the importance of respiratory PPE in settings where formaldehyde vapour cannot be fully eliminated by engineering controls.

To minimise overfitting given the limited number of outcome events, we restricted the final multivariable model to a parsimonious set of key predictors and did not include highly collinear or redundant variables for PPE (e.g., glove used in addition to face mask use because most respondents who wore face masks also wore gloves). Nevertheless, gloves remain an essential component of laboratory safety for preventing dermal exposure to formaldehyde and other hazardous chemicals. Their effectiveness depends on the material used as powdered latex gloves—commonly used in some settings—may themselves provoke irritation or allergic reactions (6). Ensuring the availability of high-quality, chemically resistant gloves remains an important preventive measure. Notably, PPE should be complemented with other control measures, such as improvements to workplace conditions, proper ventilation systems and adherence to safe-handling practices, to effectively minimise formaldehyde exposure in laboratory environments (38–40). Collectively, these strategies can substantially mitigate the risks associated with formaldehyde exposure and promote safer laboratory working conditions.

### Limitations

Several limitations should be considered. First, the cross-sectional design precludes causal inference because exposure and symptom occurrence were measured simultaneously. Second, ambient formaldehyde concentrations were not measured, preventing direct correlation between ambient levels and reported symptoms. Third, the symptoms were self-reported and may be subject to recall or perception bias. Fourth, although multiple predictors were adjusted for, residual confounding cannot be excluded, particularly from unmeasured factors, such as task variability, ventilation conditions at the time of exposure, and concomitant exposure to other gaseous pollutants. Finally, the sample size limited the number of variables that could be included in the multivariable model; thus, the findings may not be fully generalisable to other laboratories or populations.

## Conclusion

Our findings indicated that students and laboratory assistants working in the studied university laboratories were at risk of formaldehyde exposure, with one-third of the respondents reporting work-related clinical symptoms. The multivariable analysis identified several vulnerable groups, particularly women, individuals aged ≥ 30 years, and those with a history of allergies, who were at significantly higher risk of experiencing adverse symptoms of formaldehyde exposure. Frequent handling of formaldehyde further increased this risk, whereas the use of face masks provided strong protective effects.

Despite the well-documented toxicity of formaldehyde and its recognised irritant and carcinogenic properties, cadaver dissection and biological specimen handling remain integral to anatomy and laboratory training, limiting opportunities for complete substitution. Therefore, institutions must prioritise strategies to minimise exposure, such as enforcing strict adherence to hazard control measures, ensuring adequate ventilation and properly functioning exhaust systems, and enforcing consistent use of appropriate PPE, particularly respiratory protection.

Formaldehyde exposure can arise not only during active handling of formalin or treated materials but also from inadequate storage practices and poorly functioning local exhaust ventilation systems (40). Given that indoor formaldehyde concentrations in laboratory settings often exceed those measured outdoors, targeted exposure-reduction strategies are essential when elimination is not feasible. Strengthening engineering controls, improving safe-handling protocols, and enhancing training and monitoring systems will be critical to protecting laboratory personnel from the acute health effects associated with formaldehyde exposure.

## Figures and Tables

**Table 1:** Respondents’ sociodemographic, medical, and occupational history characteristics (*N* = 157)

Variables	Faculty of Medical and Health Science Laboratory (*n* = 106)	Borneo Marine Research Institute Laboratories (*n* = 51)				

	Anatomy Laboratory *n* (%)	Algae Culture Laboratory *n* (%)	Biotechnology Laboratory *n* (%)	Ecosystem Tropical Laboratory *n* (%)	Fish Disease Research Laboratory *n* (%)	Physical Oceanography Laboratory *n* (%)
**Gender**						

Male	38 (24.2)	5 (3.2)	4 (2.5)	5 (3.2)	7 (4.5)	3 (1.9)
Female	68 (43.3)	3 (1.9)	3 (1.9)	7 (4.5)	8 (5.1)	6 (3.8)

**BMI**						

Obese	8 (5.1)	1 (0.6)	0 (0.0)	1 (0.6)	2 (1.3)	3 (1.9)
Nonobese	98 (62.4)	7 (4.5)	7 (4.5)	11 (7.0)	13 (8.3)	6 (3.8)

**Age group**						

≤ 29 years	103 (65.6)	2 (1.3)	3 (1.9)	11 (7.0)	11 (7.0)	5 (3.2)
30 to 49 years	3 (1.9)	6 (3.8)	3 (1.9)	1 (0.6)	3 (1.9)	4 (2.5)
≥ 50 years	0 (0.0)	0 (0.0)	1 (0.6)	0 (0.0)	1 (0.6)	0 (0.0)

**Ethnicity**						

Bumiputera Sabah	56 (35.7)	7 (4.5)	6 (3.8)	8 (5.1)	11 (7.0)	9 (5.7)
Malay	23 (14.6)	0 (0.0)	1 (0.6)	2 (1.3)	3 (1.9)	0 (0.0)
Chinese	9 (5.7)	0 (0.0)	0 (0.0)	1 (0.6)	1 (0.6)	0 (0.0)
Indian	13 (8.3)	0 (0.0)	0 (0.0)	1 (0.6)	0 (0.0)	0 (0.0)
Other	5 (3.2)	1 (0.6)	0 (0.0)	0 (0.0)	0 (0.0)	0 (0.0)

**Alcohol consumption**						

Yes	15 (9.6)	2 (1.3)	3 (1.9)	3 (1.9)	2 (1.3)	1 (0.6)
No	91 (58.0)	6 (3.8)	4 (2.5)	9 (5.7)	13 (8.3)	8 (5.1)

**Smoking status**						

Yes	6 (3.8)	1 (0.6)	1 (0.6)	0 (0.0)	2 (1.3)	3 (1.9)
No	100 (63.7)	7 (4.5)	6 (3.8)	12 (7.6)	13 (8.3)	6 (3.8)

**Allergies**						

Yes	32 (20.4)	2 (1.3)	5 (3.2)	7 (4.5)	4 (2.5)	4 (2.5)
No	74 (47.1)	6 (3.8)	2 (1.3)	5 (3.2)	11 (7.0)	5 (3.2)

**Preexisting illness**						

Yes	26 (16.6)	3 (1.9)	2 (1.3)	6 (3.8)	1 (0.6)	2 (1.3)
No	80 (51.0)	5 (3.2)	5 (3.2)	6 (3.8)	14 (8.9)	7 (4.5)

**Job title**						

Student	102 (65.0)	1 (0.6)	2 (1.3)	10 (6.4)	11 (7.0)	5 (3.2)
Staff	4 (2.5)	7 (4.5)	5 (3.2)	2 (1.3)	4 (2.5)	4 (2.5)

**Years of study/employment at a current workstation**						

≤ 1 year	88 (56.1)	2 (1.3)	3 (1.9)	7 (4.5)	3 (1.9)	0 (0.0)
2 to 5 years	16 (10.2)	2 (1.3)	1 (0.6)	4 (2.5)	7 (4.5)	7 (4.5)
≥ 6 years	2 (1.3)	4 (2.5)	3 (1.9)	1 (0.6)	5 (3.2)	2 (1.3)

**Table 2:** Prevalence of symptoms of formaldehyde exposure by laboratory and overall (*N* = 157)

Symptoms	Medical laboratory *n* = 106 *n* (%)	Marine laboratories *n* = 51 *n* (%)	Total *N* = 157 *n* (%)	95% CI: for total % (exact)
≥ 1 formaldehyde-related symptom (any)	38 (35.8)	16 (31.4)	54 (34.4)	27.0, 42.4
Red eyes	7 (6.6)	3 (5.9)	10 (6.4)	3.1, 11.4
Itchy skin	4 (3.8)	4 (7.8)	8 (5.1)	2.2, 9.8
Sore/dry throat	2 (1.9)	1 (2.0)	3 (1.9)	0.4, 5.5
Cough	2 (1.9)	0 (0.0)	2 (1.3)	0.2, 4.5
Shortness of breath	4 (3.8)	1 (2.0)	5 (3.2)	1.0, 7.3
Chest tightness	1 (0.9)	1 (2.0)	2 (1.3)	0.2, 4.5
Wheeze	2 (1.9)	0 (0.0)	2 (1.3)	0.2, 4.5
Stuffy/runny nose	1 (0.9)	1 (2.0)	2 (1.3)	0.2, 4.5
Sneezing	0 (0.0)	1 (2.0)	1 (0.6)	0.0, 3.5
Dizziness/headache	23 (21.7)	7 (13.7)	30 (19.1)	13.3, 26.1
Stress/irritability	5 (4.7)	6 (11.8)	11 (7.0)	3.5, 12.2
Unusual fatigue/tiredness	15 (14.2)	4 (7.8)	19 (12.1)	7.4, 18.3
Pain/stiffness in neck or shoulders	6 (5.7)	3 (5.9)	9 (5.7)	2.7, 10.6
Poor memory/concentration	4 (3.8)	1 (2.0)	5 (3.2)	1.0, 7.3
Feeling depressed	1 (0.9)	0 (0.0)	1 (0.6)	0.0, 3.5

CI = confidence interval; Respondents could report multiple symptoms; Percentages are based on the total number of respondents in each group (Medical vs. Marine) or overall; Data are presented as *n* (%); 95% CI = 95% confidence interval calculated using the exact binomial (Clopper–Pearson) method

**Table 3:** Bivariable (simple) logistic regression for factors associated with adverse symptoms of formaldehyde exposure (*N* = 157)

Variables	OR	95% CI	*P*-value
**Gender**			

Male b (*n* = 62)			
Female (*n* = 95)	2.49	1.21, 5.13	0.013

**Age group**			

≤29 years b (*n* = 135)			
≥30 years (*n* = 22)	1.38	0.55, 3.48	0.489

**Alcohol consumption**			

No b (n = 131)			
Yes = 26	1.24	0.52, 2.95	0.633

**Smoking status**			

No b (*n* = 144)			
Yes (*n* = 13)	1.21	0.38, 3.90	0.748

**Allergy history**			

No b (*n* = 103)			
Yes (*n* = 54)	2.20	1.11, 4.39	0.024

**Preexisting illness**			

No b (*n* = 117)			
Yes (*n* = 40)	2.12	1.02, 4.43	0.045

**Job title**			

Staff b (*n* = 26)			
Student (*n* = 131)	1.22	0.49, 3.02	0.670

**Faculty/institute**			

Medicine b (*n* = 106)			
Marine Research (*n* = 51)	1.22	0.60, 2.49	0.581

OR = odds ratio; CI = confidence interval;

b The reference category

**Table 4:** Bivariable (simple) logistic regression for hazard control practices and adverse symptoms of formaldehyde exposure (*N* = 157)

Variables	OR	95% CI	*P*-value
**Frequency of formaldehyde handling and exposure**			

Monthly b (*n* = 67)			
Fortnightly (*n* = 47)	3.63	1.60, 8.25	0.002
Weekly (*n* = 43)	2.48	1.06, 5.79	0.036

**Average duration for each formaldehyde exposure and handling**			

< 1 h b (*n* = 64)			
1 to 3 h (*n* = 72)	2.65	1.23, 5.71	0.013
> 3 h (*n* = 21)	5.23	1.82, 15.06	0.002

**Fume hood**			

No b (*n* = 103)			
Yes (*n* = 54)	0.82	0.41, 1.65	0.578

**Localised ventilation**			

No b (*n* = 86)			
Yes (*n* = 71)	1.20	0.62, 2.32	0.594

**Biosafety cabinet**			

No b (*n* = 93)			
Yes (*n* = 64)	1.26	0.65, 2.46	0.497

**Exhaust fan**			

No b (*n* = 64)			
Yes (*n* = 93)	0.50	0.26, 0.98	0.042

**Gloves**			

No b (*n* = 62)			
Yes (*n* = 95)	0.11	0.05, 0.24	< 0.001

**Face mask**			

No b (*n* = 55)			
Yes (*n* = 102)	0.07	0.03, 0.16	< 0.001

**Chemical goggles/face shield**			

No b (*n* = 134)			
Yes (*n* = 23)	0.48	0.17, 1.38	0.273

**Laboratory coat**			

No b (*n* = 13)			
Yes (*n* = 144)	6.99	0.88, 55.27	0.265

**Closed-toe shoes**			

No b (*n* = 11)			
Yes (*n* = 146)	2.49	0.52, 11.96	0.255

OR = odds ratio; CI = confidence interval;

b The reference category

**Table 5:** Multiple logistic regression analysis of predictors of symptoms of formaldehyde exposure (*n* = 157)

Predictor	aOR	95% CI	*P*-value
Gender (female vs. male)	3.56	1.26, 10.10	0.017
Age group (≥ 30 vs. ≤ 29)	9.08	2.33, 35.35	0.001
Allergy history (Yes vs. No)	2.80	1.09, 7.25	0.033
Frequency of exposure			0.004*
Fortnightly vs. monthly	7.76	2.31, 26.09	< 0.001
Weekly vs. monthly	3.32	1.05, 10.52	0.041
Mask use (yes vs. no)	0.04	0.01, 0.10	< 0.001

* Overall *P*-value for the exposure frequency variable (three-level categorical);

aOR = adjusted odds ratio; CI = confidence interval

## References

[1] Tiruneh C (2021). Acute adverse effects of formaldehyde-treated cadavers on medical students and anatomy staff in the dissection hall at Wollo University, Northeast Ethiopia. Adv Med Educ Pract.

[2] Nisa G, Ahmad Shah B, Shahdad PS, Jan N, Samoon S, Ahmad S (2016). Acute toxic effects of formalin on first-year MBBS students during dissection in gross anatomy laboratory. IOSR J Dent Med Sci.

[3] Jangde S, Arya R, Basan K, Kumar N (2015). Preventive measures and suggested guidelines to reduce the chemical health hazards on medical students and educators. Res J Pharmacol Pharmacodyn.

[4] Zain SMSM, Azmi WNFW, Veloo Y, Shaharudin R (2019). Formaldehyde exposure, health symptoms and risk assessment among hospital workers in Malaysia. J Environ Prot.

[5] Foroughi P, Golbabaei F, Sadeghi-Yarandi M, Yaseri M, Fooladi M, Kalantary S (2024). Occupational exposure, carcinogenic and non-carcinogenic risk assessment of formaldehyde in the pathology laboratories of hospitals in Iran. Sci Rep.

[6] Alnagar FA, Shmela ME, Alrtib AM, Benashour FM, Buker AO, Abdalmula AM (2018). Health adverse effects of formaldehyde exposure to students and staff in gross anatomy. Int J Sci Res Manag.

[7] Tesfaye S, Hamba N, Gerbi A, Negeri Z (2021). Occupational formaldehyde exposure linked to increased systemic health impairments and counteracting beneficial effects of selected antioxidants. Alexandria J Med.

[8] Barile FA (2019). Alcohols and aldehydes. Barile’s Clinical Toxicology.

[9] Lemeshow S, Hosmer DW, Klar J, Lwanga SK (1990). Adequacy of sample size in health studies.

[10] Ya’acob HS, Suis AJ, Awang N, Sahani M (2013). Exposure assessment of formaldehyde and its symptoms among anatomy laboratory workers and medical students. World Appl Sci J.

[11] Vimercati L, Carrus A, Martino T, Galise I, Minunni V, Caputo F (2010). Formaldehyde exposure and irritative effects on medical examiners, pathologic anatomy post-graduate students and technicians. Iran J Public Health.

[12] Onyije FM, Avwioro OG (2012). Excruciating effect of formaldehyde exposure to students in gross anatomy dissection laboratory. Int J Occup Environ Med.

[13] Elshaer NSM, Mahmoud MAE (2017). Toxic effects of formalin-treated cadaver on medical students, staff members, and workers in the Alexandria Faculty of Medicine. Alexandria J Med.

[14] British Thoracic Society (2022). BTS clinical statement on occupational asthma. Thorax.

[15] Agency for Toxic Substances and Disease Registry (ATSDR) (2010). Addendum to the toxicological profile for formaldehyde [Internet].

[16] Abdu H, Kinfu Y, Agalu A (2014). Toxic effects of formaldehyde on the nervous system. Int J Anat Physiol.

[17] Wang HX, Li HC, Lv MQ, Zhou DX, Bai LZ, Du LZ (2015). Associations between occupation exposure to formaldehyde and semen quality: a primary study. Sci Rep.

[18] Kilburn KH, Seidman BC, Warshaw R (1985). Neurobehavioral and respiratory symptoms of formaldehyde and xylene exposure in histology technicians. Arch Environ Health.

[19] Van Thriel C, Boyes WK (2022). Neurotoxicity of organic solvents: an update on mechanisms and effects. Adv Neurotoxicol.

[20] Masnavieva LB, Efimova NV, Kudaeva IV, Zhurba OM (2023). Gender differences in sensitization to formaldehyde among urban adolescents in relation to parental chemical exposure. Hum Ecol.

[21] Whitacre CC, Reingold SC, O’Looney PA, Blankenhorn E, Brinley F, Collier E (1999). A gender gap in autoimmunity. Science.

[22] Ufelle AC, Forrest K, Smith T, Asfaw H (2022). Health effects and factors affecting formaldehyde exposure among students in a cadaver laboratory. J Environ Health.

[23] Bailey KL (2022). Ageing diminishes mucociliary clearance of the lung. Adv Geriatr Med Res.

[24] National Academies Press (US) (1987). Principles of toxicology in the context of aging. Aging in Today’s Environment [Internet].

[25] Letellier N, Gutierrez LA, Pilorget C, Artaud F, Descatha A, Ozguler A (2022). Association between occupational exposure to formaldehyde and cognitive impairment. Neurology.

[26] Bentayeb M, Billionnet C, Baiz N, Derbez M, Kirchner S, Annesi-Maesano I (2013). Higher prevalence of breathlessness in elderly exposed to indoor aldehydes and VOCs in a representative sample of French dwellings. Respir Med.

[27] Pietrzyk Ł, Torres A, Denisow-Pietrzyk M, Maciejewski R, Torres K (2016). Formaldehyde-related clinical symptoms reported by medical students during gross anatomy cadaver dissection. Dermatol Rev.

[28] Yoo Y, Perzanowski MS (2014). Allergic sensitization and the environment: latest update. Curr Allergy Asthma Rep.

[29] Hisamitsu M, Okamoto Y, Chazono H, Yonekura S, Sakurai D, Horiguchi S (2011). The influence of environmental exposure to formaldehyde in nasal mucosa of medical students during cadaver dissection. Allergol Int.

[30] Takahashi S, Tsuji K, Fujii K, Okazaki F, Takigawa T, Ohtsuka A (2007). Prospective study of clinical symptoms and skin test reactions in medical students exposed to formaldehyde gas. J Dermatol.

[31] Yu L, Wang B, Cheng M, Yang M, Gan S, Fan L (2020). Association between indoor formaldehyde exposure and asthma: a systematic review and meta-analysis of observational studies. Indoor Air.

[32] Kim YM, Kim J, Ha SC, Ahn K (2021). Harmful effect of indoor formaldehyde on atopic dermatitis in children: a longitudinal study. Allergy Asthma Immunol Res.

[33] Ahmed Y, Abdelsabour-Khalaf M, Abdelrahim E, Ghallab A (2020). Toxic effects of formalin on the medical students of South Valley University following repeated exposure at the anatomy laboratories. SVU Int J Vet Sci.

[34] Aziz MHA, Metwally EA, Ibrahim EZ, Zaki A, Azzaz OA, Hussein HA (2021). Is duration of exposure a determinant factor for genotoxicity and clinical manifestations induced by formaldehyde?. Ain Shams J Forensic Med Clin Toxicol.

[35] Zhang Q, Yan W, Bai Y, Zhu Y, Ma J (2014). Repeated formaldehyde inhalation impaired olfactory function and changed SNAP25 proteins in olfactory bulb. Int J Occup Environ Health.

[36] Wei CN, Harada K, Ohmori S, Wei QJ, Minamoto K, Ueda A (2007). Subjective symptoms of medical students exposed to formaldehyde during a gross anatomy dissection course. Int J Immunopathol Pharmacol.

[37] Mirabelli MC, Holt SM, Cope JM (2011). Anatomy laboratory instruction and occupational exposure to formaldehyde. Occup Environ Med.

[38] D’Ettorre G, Caroli A, Mazzotta M (2021). Minimizing formaldehyde exposure in a hospital pathology laboratory. Work.

[39] Ogawa M, Kabe I, Terauchi Y, Tanaka S (2019). A strategy for the reduction of formaldehyde concentration in a hospital pathology laboratory. J Occup Health.

[40] Cammalleri V, Pocino RN, Marotta D, Protano C, Sinibaldi F, Simonazzi S (2022). Occupational scenarios and exposure assessment to formaldehyde: a systematic review. Indoor Air.

